# Patient-centred care: The patients’ perspective – A mixed-methods pilot study

**DOI:** 10.4102/phcfm.v12i1.2390

**Published:** 2020-10-09

**Authors:** Roseanne E. Turner, Elize Archer

**Affiliations:** 1 Centre for Health Professions Education, Faculty of Medicine and Health Sciences, Stellenbosch University, Cape Town, South Africa

**Keywords:** empathy, patient-centred care, healthcare clinics, education, primary health care

## Abstract

**Background:**

Patient centredness is a broad concept, a moral philosophy. Patient-centred care can be viewed as the actions of patient-centredness. One of the most pertinent actions that a healthcare practitioner can utilise to deliver patient-centred care is empathic communication. Whilst many medical programmes include empathetic communication skills as part of their curricula, the recipients of this care are not asked about the relevance of this teaching.

**Aim:**

We attempted to determine whether the Western constructs of empathy were relevant in our context and also establish whether there were any parts of the medical interview which participants felt were especially important to be communicated to in their home language.

**Setting:**

Two urban communities within the City of Cape Town, Western Cape Province, South Africa.

**Methods:**

This was a mixed-methods pilot study using an explanatory sequential design. Participants who would typically make use of public health care facilities and whose first language was Afrikaans or isiXhosa were conveniently sampled. A subgroup of participants was invited to take part in a follow-up focus group discussion to add clarity to the survey responses.

**Results and Conclusion:**

Western constructs for empathy appeared to be relevant within our multicultural context. Patients wanted to communicate with their doctors and understand the cause of their problems as well as the management plan. Finally, whilst the numbers in this pilot study were too small to be generalisable, it was evident that patient-centred care was not perceived to be implemented in some public healthcare facilities attended by the participants, which resulted in them feeling unseen and disrespected.

## Introduction

The structure, content and delivery of undergraduate medical curricula have undergone important transformations over the last two decades. Medical education curricula in many institutions now include formal training in communication and language skills, and they have a focus on patient-centred care.^1^ Patient-centred medical care is important as it improves relationships between healthcare providers and their patients as well as improving health outcomes and reducing costs.^2,3^

Patient centredness is a multifaceted construct, which requires a change in doctor perspective from disease to whole-patient focus. In patient-centred care, it is the patient, not the doctor, who controls the relationship, communication and decision-making.^4^ Whilst patient centeredness is a broad concept and a moral philosophy, patient-centred care can be viewed as the actions of patient centeredness.^5^ One of the most important aspects of patient-centred care is empathic communication,^6^ which allows for effective perspective taking.

Empathy or the capacity to understand and share the feelings of another is a widely used term, with multiple meanings. Within the doctor–patient relationship, both the affective and cognitive domains of empathy are considered important.^3^ Affective or emotional empathy is an evolutionary response to emotion in others which is thought to originate from a system of mirror neurons. Affective empathy can result in emotional contagion and overidentification with others. Cognitive empathy refers to the ability to recognise and compensate for egocentricity and attempt to imagine the perspective of others. The centre for cognitive empathy lies within the cerebral cortex.^7^ Cognitive empathy is an important skill which, within the medical context, includes attempts by the healthcare provider to imagine a patient’s perspective and reflect these assumptions and understanding to the patients^3^ so patients feel understood and included in the healing process.

Patient-centred care is foundational for the delivery of culturally competent healthcare with the potential to reduce racial and ethnic disparities.^8^ Empathy in medical care is associated with many benefits including improved patient satisfaction, fewer medical errors and medico-legal claims.^9,10^

The ability to communicate effectively is an important component cognitive empathy and patient-centred care and one which needs attention in multilingual societies^11^ because, like empathy, effective communication impacts adherence and patient outcomes. According to the seminal work on communication by Albert Mehrabian,^12^ most information exchanged between people falls into the non-verbal category (voice, tone and body language), with only 7% of the spoken word being essential for communication. Thus, effective communication does not simply rely on what is said but also on how the message is expressed and the subtle interplay between verbal and non-verbal factors.^13^ More recently, Riess^14^ highlights affective empathy and enhanced non-verbal communication skills as an important component of effective medical communication.

In South Africa, which has 11 official spoken languages, patients come from a large number of different cultural groups. There have been anecdotal concern that cultural differences in non-verbal communication maybe even more challenging for the healthcare provider. The limited tested models for non-verbal empathic communication within the healthcare setting are mostly based on Western (European and American) models of empathy and may not be culturally appropriate in our more complex context. The authors were keen to explore local patient perceptions of empathy and language used by doctors to develop greater insights into this important topic.

## Aim of this pilot study

The aims of this pilot study were firstly to explore the extent to which accepted components of empathic communication developed in the West are relevant within the South African context. Secondly, we wanted to determine whether there were any parts of the clinical interview which patients perceived as more important than others about spoken language proficiency.

## Research methods and design

### Study design

**FIGURE 1:**
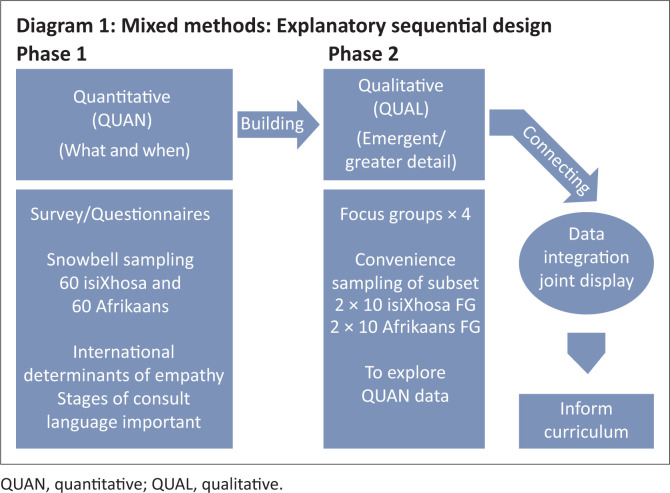
Explanatory sequential design.

This study uses a mixed-methods approach. Mixed-methods research is a relatively new research design that is more than the simple combination of qualitative (QUAL) and quantitative (QUAN) methods^15^ as it requires integration at all levels, including integration of the philosophical and theoretical lenses. In this study, an explanatory sequential design^16^ was used, which allows for the integration of population sampling, data collection and analysis. During the first phase, a positivist (QUAN) approach was used to gather and analyse data and determine the relevance of internationally reported components of medical empathy within the South African context. Following analysis of these findings, a semi-structured interview schedule was developed to guide the focus group discussions and collection of data for the second phase. These focus group discussions allowed for a deeper understanding of data highlighted in the first phase, through the use of an interpretive (QUAL) approach. The findings from both phases were integrated to ensure a deeper, synergistic understanding of the responses and are presented in a joint display. The design is illustrated in Figure 1.

### Setting and study population

The study was conducted in the Western Cape Province of South Africa where, in terms of the language policy of the Provincial Government,^17^ three official languages are required to be used and promoted. These languages are Afrikaans, isiXhosa and English. The English-language speakers were not included as English is the primary language used within the healthcare setting. The objective was to include a group of people who were not currently patients but who were from the same communities to whom medical students would be exposed. We did not sample participants at the healthcare facilities to minimise the possibility of bias. People in these communities predominately speak Afrikaans and isiXhosa. For Phase 1 of the study, a sample of 120 adults whose first language was either Afrikaans or isiXhosa were recruited, using a non-probability sampling technique from areas within the City of Cape Town located in the catchment area of the teaching hospital attached to the medical school. For the focus group discussions, a subset of 40 participants (20 from each language group) was conveniently sampled from the initial group of participants and invited to participate in one of two focus group discussions per language. Those selected needed to have completed the earlier questionnaires and be contactable via the cell phone.

### Data collection

The specifically designed questionnaire was used for phase 1 and comprised 14 items. Each item is an internationally accepted element of clinical empathy drawn from previously tested and published scales that explored empathy from the patient’s perspective. The scales included were the Patient Perception of Clinical Empathy Score (Hojat et al. 2010),^31^ the E.M.P.A.T.H.Y. tool^14^ and the CARE measure.^18^ Thus, elements of clinical empathy that appeared across all these scales were consolidated and presented as a 5-item Likert-type scale. Likert scales are rating scales, which in this study required participants to indicate how important each item was in making them ‘feel your doctor understands and cares about you’. Five possible options (from 1 to 5) along a continuum are as follows: 1 = ‘not at all important’, 2 = ‘slightly important’, 3 = ‘neutral’, 4 = ‘quite important’ and 5 = ‘extremely important’. The reason for including these established items was to determine whether the elements of empathic communication are consistent and relevant across diverse cultures in South Africa.

This was followed by a second subset of questions using a similar scale. These questions listed the stages of the doctor visit as described in the Calgary Cambridge guide, to the medical interview which is taught to the medical students at our institution.^19^ In this section, participants were asked to rate, ‘how important is it that your doctor speak your home language well during the following stages of your visit’.

At the time of data collection, there was an increase in gang violence within the target community, necessitating the deployment of the South African Defence Force. Concern for researcher and participant safety influenced sampling decisions. Some participants were sampled outside shopping malls in two Cape Town suburbs and invited to complete the questionnaires. The IsiXhosa participants were recruited at school functions and access to further participants gained through community gatekeepers. Consent was signed and participants were provided with an explanation about how to complete Likert scales before being asked to complete a questionnaire in their home language. Translated questionnaires were available in both Afrikaans and isiXhosa. Data collection occurred during weekday afternoons.

Following the analysis of the first phase, an interview schedule was developed to allow further exploration of aspects of non-verbal communication essential for the development of trust indicative of care. Also, preferences around the use of interpreters to convey meaning were explored, if doctors are not proficient in patient’s home language.

Both Afrikaans focus groups were conducted in the same venue at different times. Because of safety and other logistical concerns, the private home of a member of the community had been offered, which was considered safe, central and easily accessible for all participants. At the time of the study, gang violence in this area was of particular concern. Two isiXhosa focus groups were conducted in a meeting hall in the community library. There were two first-language Afrikaans or isiXhosa speakers and facilitators for each of the focus groups. These facilitators were all part of the research team. A copy of the semi-structured interview schedule used, has been attached.

### Data analysis and integration

In mixed-methods research, data obtained during the QUAN and QUAL phases must be integrated and presented as a whole and in such a way that it allows greater understanding of the findings than when each aspect was reported independently.^20,21^

The QUAN survey data were entered into an Excel spreadsheet for simple descriptive analysis.

Recordings from four focus groups were translated into English and then transcribed. The transcriptions were then thematically coded by two researchers. The first researcher coded inductively by hand, whilst the second used Atlas.ti (V8), a qualitative data computer analysis software (QDCAS) package as a form of triangulation.^20^ This article reports on the QUAN findings (questionnaires) together with some quotes from the QUAL data (focus group interviews). The integrated data are presented as a joint display.^21,22^

### Ethical consideration

The protocol for this project (HREC Reference # N19/01/009) was reviewed by the Stellenbosch University Health Research Ethics Committee 2 (HREC2) and approved on 08/04/2019.

## Results

Questionnaires were completed by 120 participants; 60 first-language isiXhosa speakers and 60 first-language Afrikaans speakers. All those who were approached agreed to participate.

### Demographic data

**FIGURE 2:**
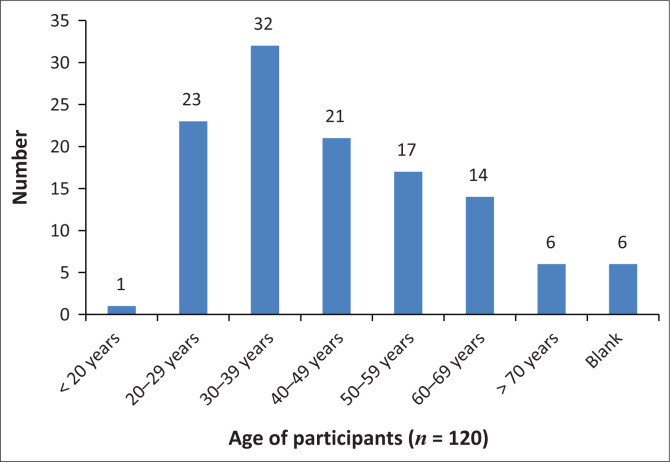
Participant age.

**FIGURE 3:**
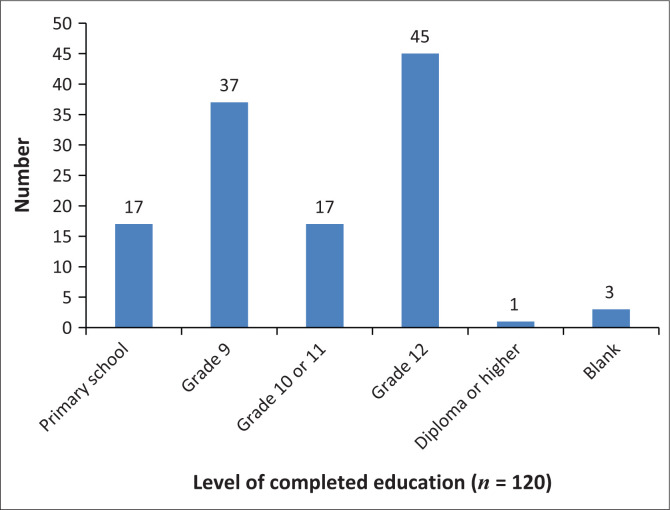
Level of education.

**FIGURE 4:**
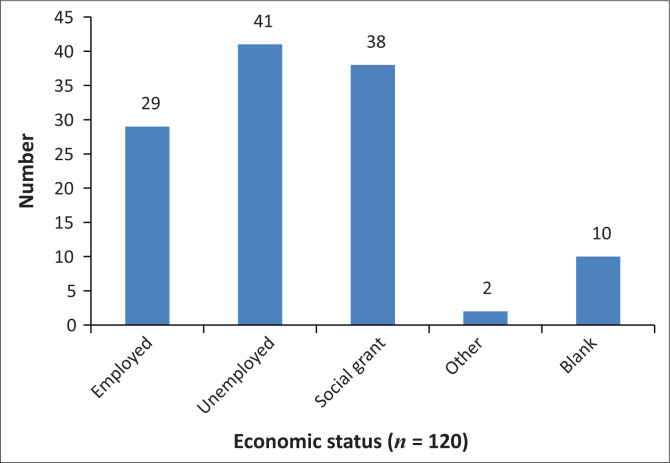
Economic status.

Demographic data that aimed to capture details about age, gender, level of education, economic status, frequency of visits to the doctor and whether doctors were consulted in the state or private sector were included. During conceptualisation, we had hoped to gain insight into whether the expectations of empathy varied based on education, economic status and those using public- or private-sector healthcare. This was not possible because of logistical issues; and in retrospect, the demographic section of the questionnaire should have been simpler and clearer as only the questions related to age, level of education and economic status (Figures 2–4) were adequately completed by the participants and able to be analysed and presented.

The youngest participant was 19 years old and the oldest 84 years. Five participants were in their 70s and six did not complete this section.

Almost 70% of participants had not completed Grade 12, and one participant had a higher education qualification. The data are probably not representative of the population in the area as sampling occurred during weekday afternoons when those who are employed as well as those at higher education institutions would not be available. A small percentage (25%) of participants sampled was employed. However, because the majority of participants were unemployed and receiving social grants, they were more likely to attend public hospitals and clinics when requiring healthcare.

In the section below, the results from the survey are presented (Tables 1 and 2), followed by the integration of the QUAN and QUAL data (Table 3).

**TABLE 1:** Empathy items in rank order, which indicate to the individual that the doctor understands and cares about them.

Items in rank order (high to low)	*n*	Not important (%)	Neutral (%)	Important (%)
Q01 = The doctor tries to make me feel comfortable	120	6.6	8.3	85.0
Q08 = The doctor does a proper physical examination	115	7.0	9.6	83.5
Q14 = The doctor is kind and reassuring	119	8.4	10.1	81.5
Q06 = The doctor listens to what I say without interrupting	118	10.2	9.3	80.5
Q07 = The doctor is interested in my feelings and worries	118	10.3	12.1	78.0
Q09 = The doctor can tell me what is wrong with me	116	10.3	12.1	77.6
Q04 = The doctor makes eye contact with me	117	14.5	8.5	76.9
Q02 = The doctor introduces himself or herself to me	116	20.7	5.2	74.1
Q12 = The doctor includes me in decision-making	119	14.3	12.6	73.1
Q03 = The doctor calls me by my name	114	14.9	12.3	72.8
Q10 = The doctor tries to understand the way I see things	116	13.8	20.7	66.5
Q05 = The doctor tries to speak my home language	117	23.9	15.4	60.7
Q11 = The doctor is respectful of my customs	120	31.7	15.8	52.5
Q13 = The doctor tells me about his or her own problems and challenges	116	62.1	15.5	22.4

**TABLE 2:** Required language proficiency during the clinical visit.

Language proficiency at different stages of the clinical visit	*n*	Not important (%)	Neutral (%)	Important (%)
Stage 1: On greeting and start of the visit	119	14.3	10.1	75.6
Stage 2: Information gathering	117	10.3	16.2	73.5
Stage 3: During the physical examination	116	8.6	9.5	81.9
Stage 4: Explaining and planning treatment	117	11.1	10.3	78.6
Stage 5: End of visit	119	12.6	15.1	72.3

**TABLE 3:** Integrated quantitative and qualitative data.

Top eight ranked items from the survey	Quotes from the focus group interviews that give a deeper understanding of the respective survey questions
1. The doctor tries to make me feel comfortable	‘When visiting a doctor you wish to meet someone who is friendly and welcoming because you are there to discuss your problems’.
85%	‘… You walk in and say, “Morning doctor” but the doctor is busy with the files, he doesn’t answer’.
2. The doctor does a proper physical examination	‘Doctors do not really want to examine you. They look at what is wrong with you’.
83.5%	‘In the room, there is the doctor, you sit over there at the table on the chair … he does not examine you’.
	‘We talk but the bed is over there, white and nicely made up. You have this ailment and he only looks in your file to see what is there’.
3. The doctor is kind and reassuring	‘A doctor should be someone who is friendly, so you feel comfortable talking around them’
81.5%	‘the doctor said if you don’t want to wait you should get yourself a medical aid’.
4. The doctor listens to what I say without interrupting	‘You realise the doctor isn’t interested and feels like stopping you when you are talking’
80.5%	‘They don’t listen, sometimes they look at you on the outside … and they don’t give much attention to the dirty ones’.
5. The doctor is interested in my feelings and worries	‘I do not feel like talking to one that is not interested’
78%	‘Sometimes you are busy with the doctor, and a nurse or another doctor comes in and they start having a different conversation, and the doctor continues writing in my folder while having that conversation …’
	‘I feel happy when the doctor greets me to show that she’s interested’.
6. The doctor is able to tell me what is wrong with me	‘You want him to explain the illness and its cause, instead of just giving you medicine. They should tell you so you can prevent it from happening again’.
77.6%	
7. The doctor makes eye contact with me	‘A doctor should make eye contact to see what you are talking about’.
76.9%	‘The eye contact is very important to me. Look me in the eyes, then I know we understand each other.’
8. The doctor introduces themselves to me	‘They don’t introduce themselves and they don’t wear name tags.’
74.1%	We don’t know the name. ‘The doctor with the sweater, or the one with the frizzy hair or the one who always wears a scarf on her head’
‘When they have the biggest phone you know this is the doctor’.	

### Quantitative data

As described above, participants were asked to rate the importance of various elements of cognitive empathy using a 5-point Likert rating scale. During analysis, the ranks ‘not at all important’ and ‘slightly important’ were combined and appear in Table 1 as ‘not important’. And ranks ‘quite important’ and ‘extremely important’ were combined and are presented (Table 1) as ‘important’. These items were then ranked according to the percentage of participants who considered them important. All responses over 40% are considered to be significant because the probability of any of the points on the Likert scale being selected was equal, and could have resulted in an even distribution across all five points (i.e. 20% per point).

### Language proficiency and the various stages of a clinical visit

Participants were also asked the following questions in the questionnaire: ‘How important is it to you that your doctor can speak your home language well during the following stages of your visit?’ See Table 2 for the results. Participants expressed a desire to be able to properly understand and be understood during all stages of the clinical visit. Stages 3 (the physical examination) and 4 (explaining and planning treatment) of the visit were rated as the most important stages.

### Qualitative data

We have integrated the data by linking relevant quotes from the focus group interviews (QUAL) with the survey findings (QUAN) to give the readers deeper insight.

### Integrated data

In Table 3, the data from both phases are integrated and presented in a way which allows a deeper understanding of the initial survey findings. Only the top eight ranked items in the survey are presented in the left column. These items were later explored more in-depth during the focus group discussions. Quotes from the transcripts which most clearly represent participant views about the survey questions have been included in the right column.

## Focus group findings

### Language proficiency

Language proficiency was considered important during all phases of the clinical interview. *In this section, quotes from the focus group interviews have been inserted into the text in italics.* Being able to be understood by and to understand the doctor is an important part of the clinical visit. Participants felt that doctors must be able to cater to the needs of the community. Appointing foreign doctors or those that do not speak any of the local languages well ‘*does not work for the community*’ because ‘*they cannot interact*, and you *cannot put the problem on the table*’ which means ‘*they do not understand the problem*’. Participants want to be told what is happening to them, so they can understand any ‘*changes in treatment or their condition*’, ‘*terminology*’, ‘*what is wrong with them*’ as well as ‘*what they need to do to get better*’, what the doctor plans to do and ongoing ‘*details about their condition*’. Participants expressed a need to be able to understand what is happening in their body, so they can take independent action to help themselves in the future and prevent the same situation occurring again. Whilst participants felt verbal language is important to ensure both parties understand each other, a lack of fluency was accepted if the patient felt comfortable with the doctor:

‘[*S*]he tried, she tried to use at least one Afrikaans word in a sentence. … in that case language wasn’t a problem.’

‘Even if communication is a barrier, if the person feels comfortable and trusts the doctor it will not be an issue.’ (Quotes from Focus group)

### Interpreters

Participants want to be part of the clinical process; and whilst they would prefer the doctor to be able to speak their language, they understand this is not always possible. Patients want interpreters to help them access information if the doctor does not speak their language. Indeed participants said they would be comfortable for information to be communicated through ‘*interpreters*’, ‘*sign language*’ or even ‘*cell phone applications*’. Some were concerned about the use of interpreters and the confidentiality of their information and stressed it is important to make use of interpreters who have an understanding of medical terminology.

## Discussion

To the best of our knowledge, this is one of the first South African studies exploring community perceptions specifically related to empathy and patient-centred healthcare. One of our aims was to explore the language needs of various groups in the community who may not be proficient in English. It became clear during the focus group discussions that patients often feel unseen at public clinics, regardless of what language is being spoken. This highlights a potentially more urgent need, especially given the rapidly escalating levels of medico-legal litigation in this country.^23^

Participants from two metro areas within the Northern subdistrict of the City of Cape Town were approached during working hours. The demographic data indicate levels of disempowerment, i.e. the low levels of education, high unemployment and continued socio-economic deprivation in the sampled areas. Almost three quarters (73.5%) of participants were aged under 50 years. Despite being fairly young, participants reported suffering a variety of chronic health conditions necessitating regular visits to healthcare clinics, which is another indication of socio-economic deprivation.^24^

Many examples are available in the focus group discussions, which appear to highlight that patients are very aware of these power differences, for example, the multiple references to the size, model and functionality of the doctor’s cell phone. The highest ranked needs in the QUAN data related to a desire for the doctor to make them feel comfortable and to be friendly and welcoming.

Patient empowerment is important for the improvement in medical outcomes as well as lowering treatment costs.^25^ Within our sample, there are multiple potential power differentials between patients and the doctor, which include levels of authority, environmental familiarity, education and economics. These all impact both the need for empathy (by patients) and the ability to be empathic.^7^ Riess^10^ highlights the unconscious evolutionary ability among individuals to notice differences, which may be psychologically threatening. Cubaka et al.^26^ report that patients viewed themselves as ‘lower’ than doctors, which resulted in them needing to be obedient. Riess and Kraft-Todd^17^ highlight patients’ reluctance to disagree with their doctors, which underlines the importance of effective non-verbal communication in an attempt to deliver effective patient care. The ability to quickly put patients at ease, so that they may feel safe, ‘seen’ and respected is an important component of both patient empowerment and patient-centred care.^9,10^

Most participants in this study complained about being unable to establish any relationship with their constantly changing doctors who appeared more interested in the files than in their patients. Doctors need to be able to access the patient’s story, and this fact must be conveyed to patients who appear to place more emphasis on the physical examination than on the history taking. Doctors could easily include patients in their review of patient history as a means of establishing empathic communication. Swaminath^10^ reports that patient satisfaction and treatment compliance has more to do with patients’ feeling a ‘sense of partnership’ with the doctor compared to the actual information shared. Given the many administrative and logistical challenges presented by under-resourced healthcare clinics, the ability to communicate empathically is an effective way of both improving patient outcome^27^ and reducing doctor burnout.^28^

According to Levison et al. (2010)^29^ patients assess the quality of their care based on their experience of being heard and understood by the doctor. The order in which participants ranked the empathy items in the questionnaire highlighted the subjective rather than objective ideas of empathy. This is important as it appears that patient needs could be relatively easy to meet within healthcare facilities without additional resources. The top-ranked needs were as follows: the doctor tries to make me feel comfortable (85%), the doctor does a proper physical examination (83.5%), the doctor is kind and reassuring (81.5%) and the doctor listens to what I say without interrupting (80.5%). Anecdotally doctors often express concern that this sort of interaction is not possible, given the high patient load, yet Swaminath^10^ highlights that one takes only a few minutes longer for this type of engagement. Satisfied patients have better outcomes and improved adherence to treatment and so ultimately will require fewer clinic encounters in the long run.

Patients expressed a wish to be addressed in their home language although they understood this was not always possible. They want to be able to understand and be understood especially during the physical examination and whilst treatment is being planned and explained. These findings are similar to those reported by Cubaka et al.^26^ who explored patient perceptions in Rwanda. In their study, patients said they valued being able to communicate and interact with caring, empathic providers who not only were able to share information but also involved them in their care.^26^ Patients ideally need to be actively engaged with their treatment;^30^ but for this to happen, an understanding of language is required. Whilst patients do understand that it is not always practically possible to match doctor and patient home languages, they indicated that it was the effort and intention to communicate which was important, even if the actual message is conveyed via a third party or digital technology.

## Strengths and limitations

This pilot study provided an opportunity for the voices from two communities to be heard about empathy. To research communities takes considerable time and resources and therefore we believe that whilst this is a small scale study, we have contributed towards the body of knowledge. We are not aware of previous studies where members of the community have expressed their needs and opinions related to empathic communication in healthcare clinics.

An escalation of gang violence impacted sampling and data collection. However, the authors do not believe these factors negatively influenced the outcome of this study.

This was a small pilot study and so is not representative nor generalisable. The small number of participants is, therefore, a limitation. The lessons learned from this pilot will, however, be useful in assisting us with the development of a larger follow-up study with patients.

An unexpected limitation was the low level of basic education that complicated data collection. The questionnaire and relatively complex Likert scales were not fully understood by some participants despite prior explanation from the research team.

## Implications or recommendations

- There is a need for patient-centred care in public healthcare clinics.
- The ranked list of empathy items highlights a low cost and easy intervention to increase patient satisfaction and potentially reduce medico-legal litigation, namely, training doctors in empathic communication.
- Future research is needed regarding the use of interpreters and technology to relay accurate information as an adjunct to patient-centred care.
- The perceptions of first-language English speaking as well as employed participants and adolescents would add valuable insights.

## Conclusion

The components of empathy articulated in Western research are relevant in our multicultural context. Patients want their doctors to listen and to examine them. They also want to be able to understand their doctors, so they can be more actively involved in their health. Patients want their doctor to be empathic and try to make them feel comfortable. They want doctors to be kind and respectful and to try to communicate. If these are in place then interpreters would be an acceptable way to address language deficits.

It may be possible to implement simple strategies to improve empathic communication within public healthcare clinics to ensure patients feel seen and respected.
